# A stratification method based on clustering for the minimization of data masking effect in signal detection

**DOI:** 10.1186/s12911-020-1037-z

**Published:** 2020-02-03

**Authors:** Jian-Xiang Wei, Yue Ding, Ming Li, Jun Sun

**Affiliations:** 1 School of Internet of Things, Nanjing University of Posts and Telecommunications, Nanjing, 210003 China; 2 Jiangsu Center for ADR Monitoring, Nanjing, 210002 China

**Keywords:** Adverse drug reaction, Signal detection, Data masking, Stratification, Clustering

## Abstract

**Background:**

Data masking is an inborn defect of measures of disproportionality in adverse drug reactions (ADRs) signal detection. Many previous studies can be roughly classified into three categories: data removal, regression and stratification. However, frequency differences of adverse drug events (ADEs) reports, which would be an important factor of masking, were not considered in these methods. The aim of this study is to explore a novel stratification method for minimizing the impact of frequency differences on real signals masking.

**Methods:**

Reports in the Chinese Spontaneous Reporting Database (CSRD) between 2010 and 2011 were selected. The overall dataset was stratified into some clusters by the frequency of drugs, ADRs, and drug-event combinations (DECs) in sequence. *K-means* clustering was used to conduct stratification according to data distribution characteristics. The Information Component (IC) was adopted for signal detection in each cluster respectively. By extracting ADRs from drug product labeling, a reference database was introduced for performance evaluation based on *Recall*, *Precision* and *F*-measure. In addition, some DECs from the Adverse Drug Reactions Information Bulletin (ADRIB) issued by CFDA were collected for further reliability evaluation.

**Results:**

With stratification, the study dataset was divided into 21 clusters, among which the frequency of DRUGs, ADRs or DECs followed the similar order of magnitude respectively. *Recall* increased by 34.95% from 29.93 to 40.39%, *Precision* reduced by 10.52% from 54.56 to 48.82%, while *F-*measure increased by 14.39% from 38.65 to 44.21%. According to ADRIB after 2011, 5 DECs related to *Potassium Magnesium Aspartate*, 61 DECs related to *Levofloxacin Hydrochloride* and 26 DECs related to *Cefazolin* were highlighted.

**Conclusions:**

The proposed method is effectively and reliably for the minimization of data masking effect in signal detection. Considering the decrease of *Precision*, it is suggested to be a supplement rather than an alternative to non-stratification method.

## Background

Spontaneous reporting system (SRS) is one of critical data resources for adverse drug reactions (ADRs) surveillance. Tau N et al. demonstrated that the most frequent information sources that served as the basis of the initial safety signal in the Unite States were Food and Drug Administration’s adverse event reporting system (87 [38%]) and randomized clinical trials (81[36%]) or observational studies among the 228 drug safety communications [1]. In China, medical institutions, pharmaceutical manufacturers and patients report adverse drug events (ADEs) through SRS in the voluntary reporting approach. Each report is assessed by experts of pharmacovigilance before being recorded into the Chinese Spontaneous Reporting Database (CSRD). Up to now, the number of new reports has exceeded one million per year. An important task for pharmacovigilance is to discover potential risks for postmarketing drugs by signal detection based on the CSRD. As the quality of reports varies greatly, all reports of SRS are mainly used for hypothesis generation of suspicious signals rather than evidence.

The conventional methods of ADR signal detection are mainly based on disproportionality analyses [2], such as Proportional Reporting Ratio (PRR), Reporting Odds Ratio (ROR), the integrated standard method taken by Medicines and Healthcare Products Regulatory Agency (MHRA), Information Component (IC), Multi-item Gamma Passion Shrinker (MGPS) and so on [3–11]. Although these methods have achieved acceptable performance [12, 13], they are strongly affected by several biases, such as under-reporting, misdiagnosis and selective reporting [14, 15], which may lead to data masking effect [16–18] or competition bias [15, 19].

Data masking is a collateral effect of quantitative methods in signal detection, which relies on disproportionality analysis by which signals of suspected drug-event combinations (DECs) may be delayed or hindered because of the over-reporting of another DEC [20]. The previous researches for minimizing data masking can be classified into three categories: data removal, regression and stratification method. In data removal method, some specific data such as the known DECs [19] and reports related to drug competitors [21] were removed to correct for competition bias and highlight suspected DECs of interest. Some reasonable statistical decision rules were proposed to determine the type and quantity of data to be removed more objectively [22]. Arnaud et al. identified [15] potential competitors via competition index, as well as masking factor [23] and masking ratio [20] and performed signal detection after removing reports mentioning such competitors. In general, the performance of data removal methods is highly dependent on human decision and rule-making. Different from data removal methods, Caster et al. [24, 25] applied lasso logistic regression into ADR surveillance and highlighted more DECs signals related to specific drugs earlier than the IC method. Each report was treated as observation object [16, 26] to avoid losing data, however the computation process was extremely tedious and time-consuming. Furthermore, some researchers thought that ADRs were mostly related to drugs’ medicinal properties, but the confounders of patients could not be simply ignored (e.g., age, gender, region), which would result in many false signals [11, 27, 28]. Ye et al. [29, 30] stratified the whole dataset into several strata according to suspected confounders and performed signal detection separately. However, it should be noted that confounding could only be evaluated in the absence of effect modification [28, 31], otherwise the integrity of data would be destroyed and false signals might come.

These adjusted methods for signal detection are mainly based on measures of disproportionality, in which suspected DECs signals are highlighted by disproportionate observed-to-expected (OE) ratios. The OE ratios are strongly affected by over-reported drugs or ADRs, and some specific DECs corresponding to true signals which are rarely reported may be masked with lower OE ratios. Therefore, frequency difference of ADEs is an important factor of data masking, which has not been considered in the above methods. It is reasonable to stratify the data into some clusters, among which the data is of similar order of magnitude. The aim of this study is to explore a novel stratification method to reduce the impact of frequency differences on true signals masking.

## Method

### Data source

All reports of ADEs in the CSRD between 1 January 2010 and 31 December 2011 were selected. By preprocessing, a study dataset including 1,081,898 records was obtained, which included 1763 drugs, 877 ADRs and 37,193 DECs.

A reference database was considered as the gold standard for performance evaluation, which contained ADRs extracted from drug product labeling manually. If some DECs exist in the reference database but are not detected as positive signals by disproportionation analysis, we suppose these DECs are masked. Among 37,193 DECs from the CSRD, there are 12,493 DECs existing in the reference database and we denote them as known DECs.

### Stratification strategy

Disproportionality analysis method, such as IC, is based on an OE ratio comparing the relative reporting rate of the ADR for a specific drug with that for the overall drugs in the database. If the usage quantities of drugs are equivalent, OE ratio may be more reliable. In a sense, commonly used drugs are of more reports. For example, among 1763 drugs in our study dataset, *Levofloxacin Hydrochloride* and *Azithromycin*, the two widely used drugs, were reported 111,335 times and 78,449 times, accounting for 6.18 and 4.35% of all reports respectively. Thus, the frequency of reports in SRS reflects the usage quantities of drugs indirectly.

We scanned the study dataset by the IC method and compared all suspected signals with the reference database. Table 1 revealed that the average frequency of reports on all drugs was 1022.06 times, while the average frequency of reports on the drugs related to masked DECs was 366.96 times with frequency decline rate 64.10%. The signals of drugs with less frequency were more likely to be masked, just as Maignen et al. mentioned that the strongest masking effect was associated with the drug with the highest number of records for any event [20]. Similarly, overall averages on ADRs and DECs were 2054.62 times and 48.45 times, while the corresponding average frequency of reports involved in the masked signals were 1001.76 times and 48.29 times, declined by 51.24 and 0.33% respectively. The frequency difference of drugs (64.10%) is most obvious, followed by ADRs (51.24%) and DECs (0.33%). Therefore, in order to reduce the impact of frequency difference on OE ratio, the stratification will be conducted in the sequence of “DRUGs-ADRs-DECs”.

**Table 1:** Statistics of ADE reports

	Average frequency of reports on DRUGs	Average frequency of reports on ADRs	Average frequency of reports on DECs
Related to masked DECs	366.96	1001.76	48.29
Overall average	1022.06	2054.62	48.45
Decline rate^a^	64.10%	51.24%	0.33%

^a^Decline rate: the percentage of decrease in frequency of masking signals compared to the overall average

### Stratification procedure

The stratification process can be described as follows:

- Step 1: Stratify the study dataset according to the frequency distribution characteristics of DRUGs. The frequency of ADR reports is counted for each drug and *K-means* clustering algorithm is adopted to partition the study data into several clusters. Cluster refers to a group of objects with the similar characteristic, and in this case, it refers to a group of drugs with similar order of magnitude in frequency.
- Step 2: Further divide each cluster into multiple small clusters based on the frequency of ADRs.
- Step 3: Conduct repetitive operations based on DECs subsequently to divide the study dataset into many smaller clusters.

Specifically, a cluster is divided into some small clusters in each stratification by following processes: analyze data distribution, determine the number of clusters and perform stratification with *K-means* algorithm. *K-means* is a clustering algorithm frequently used in data mining. It aims to partition *m* objects into *k* clusters, in which each object has the similar attributes. *k* is a parameter that needs to be predefined, representing the number of clusters. First, *k* cluster centers are randomly selected from all objects. The remaining objects are assigned to the different cluster based on the similarity measure between the object and all cluster centers. Then, cluster centers are updated by computing the mean of the objects in the same cluster. All objects are arranged into new clusters with this iterative refinement technique. Considering that the intensive areas in the data distribution chart will form peaks, *k* is determined by peaks number of data distribution in this study.

### Signal detection method

The IC method is adopted for signal detection. The lower limit of the 95% confidence interval is referred to as IC_025_, which is the standard measure used to screen the WHO database for excessive ADR relative reporting rates [27]. The signal with the threshold at IC_025_ > 0 is considered suspected.

### Performance evaluation

Three classic indicators are adopted for performance evaluation, including *Precision*, *Recall* and *F*-measure [32]. *Precision* is a measure of exactness, indicating the percentage of DECs labeled as positive that are actually ADRs. *Recall* is a measure of completeness, indicating the percentage of DECs corresponding to ADRs that are labeled as positive. There tends to be an inverse relationship between *Precision* and *Recall*, where it is possible to increase one at the cost of reducing another. An alternative compromise is *F-*measure, which is the harmonic mean of *Precision* and *Recall.* According to the reference database, true positive (TP) represents the number of known DECs accurately detected as positive signals, false negative (FN) represents the number of known DECs detected as negative signals, false positive (FP) represents the number of unknown DECs detected as positive signals and true negative (TN) represents the number of unknown DECs detected as negative signals. Based on TP, FP, FN and TN, the three indicators are calculated for comparing the performance differences between stratification and non-stratification.

Meanwhile, some ADEs of the Adverse Drug Reaction Information Bulletin (ADRIB) after 2011 issued by CFDA are collected for reliability evaluation.

## Results

### Stratification results

To determine the numbers of clusters in each step, statistical analysis was adopted on data distribution. In the first step of stratification, we found the frequency of different drugs varied dramatically. For example, *ω-3 Fish Oil Fat Emulsion* and *Bicalutamide* were reported only three times, while *Levofloxacin Hydrochloride* was up to 111,335 times. The statistic analysis of data distribution would not be obvious on account of the large data range. The natural logarithm (*ln*) was introduced to compress the scales. The frequency ranging from 3 to 111,335 was transformed into an interval [1.1, 11.6], and the frequency statistics was performed with an interval step of 0.5.

**Fig. 1:**
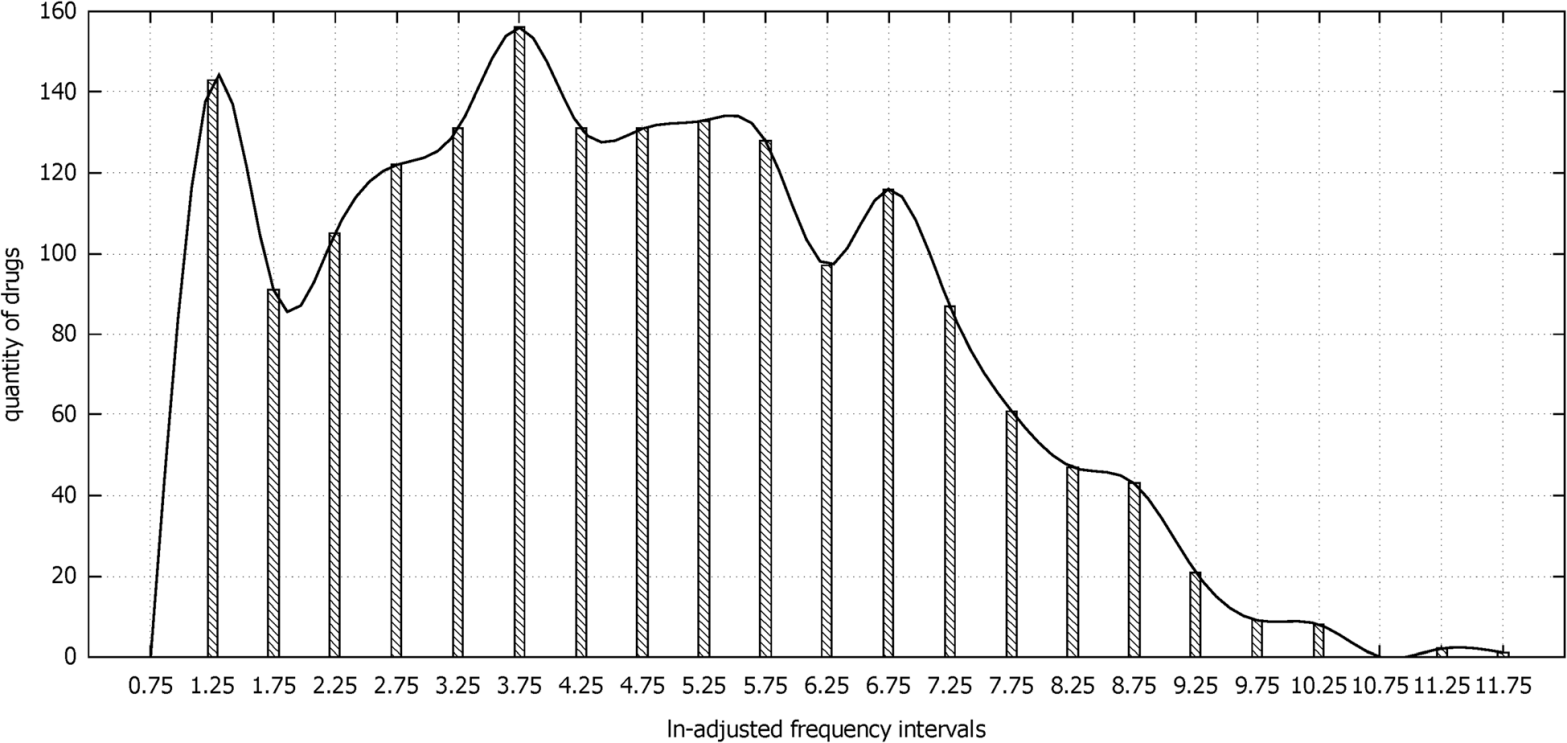
Frequency distribution of drugs

The frequency histogram of 1763 drugs was presented in Fig.1. The *ln*-adjusted frequency intervals labeled the horizontal axis, and quantity of drugs marked the vertical axis. There were three peaks in Fig. 1, and each peak indicated that the data was concentrated in corresponding magnitude range. Therefore, the cluster number based on DRUGs was set as 3, and *K-means* clustering algorithm was performed by SPSS 19.0. Similar operations were conducted based on ADRs and DECs in sequence.

**Fig. 2:**
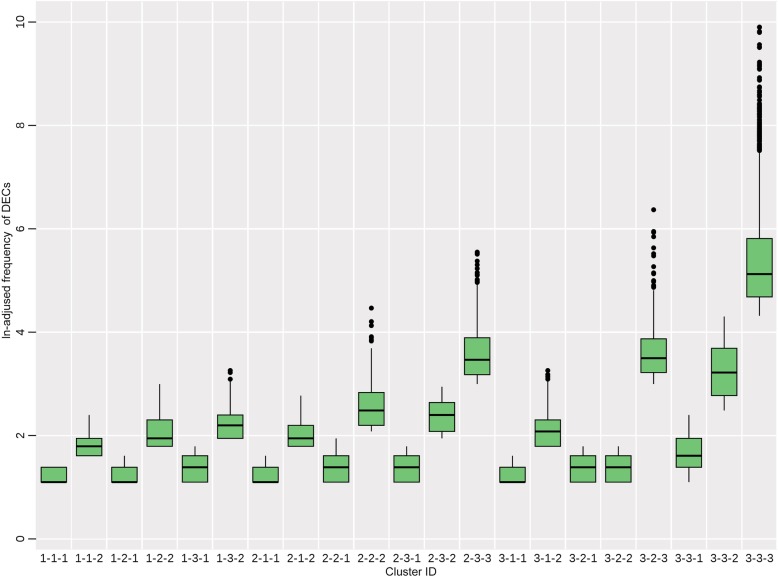
Frequency distribution of DECs in each cluster

With stratification, the study dataset was eventually divided into 21 clusters, among which the frequency of DRUGs, ADRs or DECs followed the similar order of magnitude respectively. Taking DECs as an example, the frequency distribution of each cluster was illustrated by a box and whisker diagram (Fig. 2). The vertical axis represented the *ln*-adjusted frequency of DECs. The horizontal axis represented the cluster ID, which meant the hierarchical relationship among clusters. For example, the ID 1–1-1 indicated DRUGs cluster 1 → ADRs cluster 1 → DECs cluster 1.

The maximum value and minimum value of each cluster were mostly in the similar order of magnitude. However, there were a few outliers in some clusters, such as cluster 3–2-3 and cluster 3–3-3. The reason for the existence of outliers was that the relatively low-frequency DECs occupied a large proportion.

### Performance for unmasking

Using the IC method, 6853 suspected signals including 3739 known DECs were detected with non-stratification, and 10,336 suspected signals including 5046 known DECs were detected with stratification. Detailed results were shown in Table 2, where the values in brackets were the results of non-stratification.

**Table 2:** Signal detection results of stratification and non-stratification

	Positive	Negative
	Stratification (Non-stratification)	Stratification (Non-stratification)
Known	5046 (3739)	7447 (8754)
Unknown	5290 (3114)	19,410 (21,586)

With stratification, the increase in TP was 1307, while the increase in FP was 2176. Table 3 showed that *Recall* increased by 34.95% from 29.93 to 40.39%, *Precision* reduced by 10.52% from 54.56 to 48.82% and *F-*measure increased by 14.39% from 38.65 to 44.21%. The considerable improvement of *F-*measure confirmed the effectiveness of the proposed method.

**Table 3:** Performance evaluation of stratification and non-stratification

	*Precision*	*Recall*	*F*
Non-stratification	54.56%	29.93%	38.65%
Stratification	48.82%	40.39%	44.21%

**Fig. 3:**
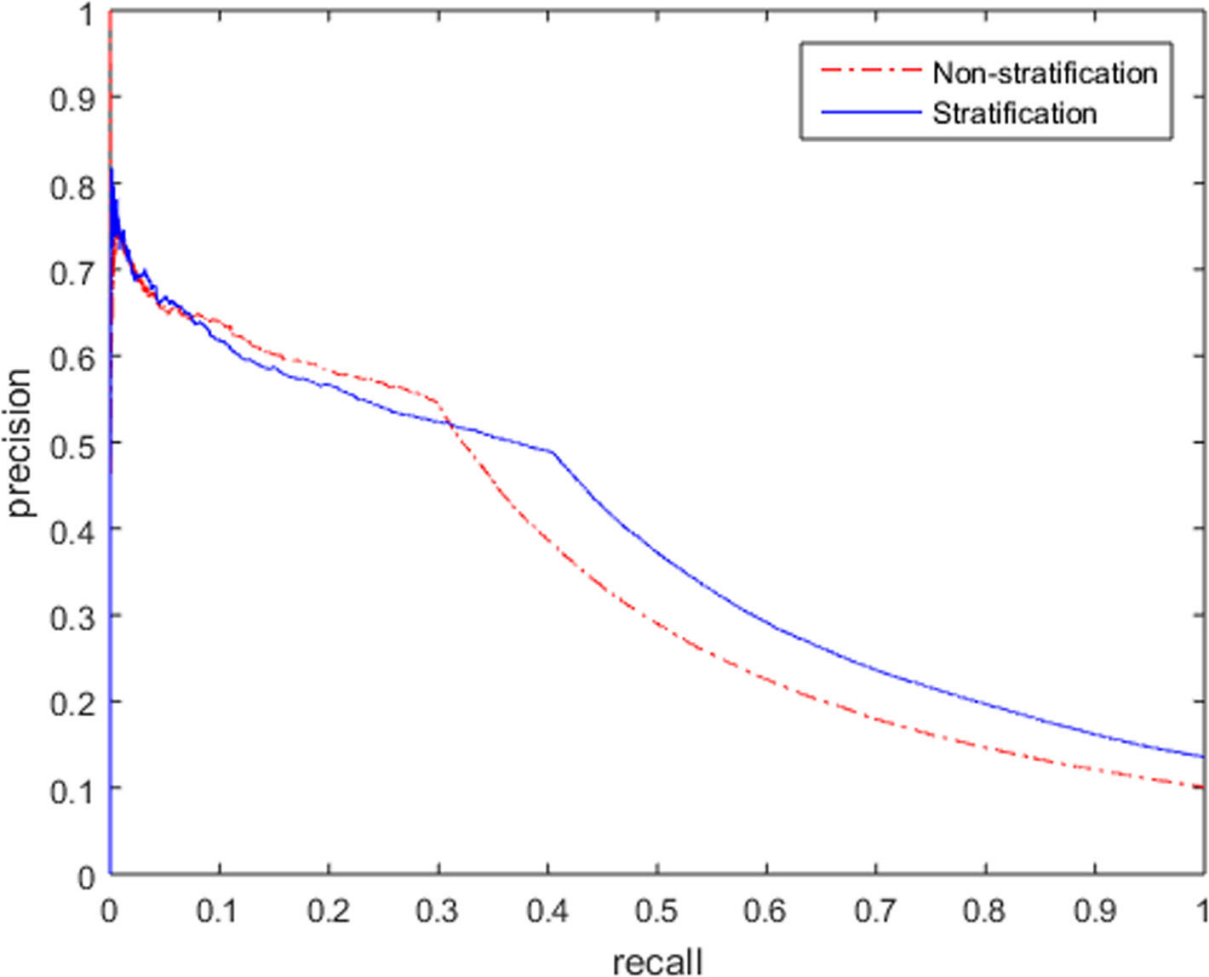
Precision-recall curve

A precision-recall curve was introduced to evaluate the overall performance of the method. The study dataset was sorted in descending order based on the IC_025_ values for stratification and non-stratification, and *Precision* and *Recall* for each DEC were calculated gradually. Figure 3 showed that *Recall* of non-stratification was slightly better than that of stratification under the same *Precision* at the beginning of signal detection. With more and more TP signals were detected subsequently, *Recall* of stratification was significantly better than that of non-stratification. On the whole, under the same *Precision*, *Recall* of stratification was better than that obtained without stratification, which proved that the performance of signal detection had improved with stratification.

For further reliability evaluation, we selected out some DECs from the ADRIB after 2011 issued by CFDA which contained true signals that were not present in the reference database. The DECs were not detected as positive signals by non-stratification, but newly highlighted by stratification. As listed in Table 4, some ADEs related to *Potassium Magnesium Aspartate* (in the 50th ADRIB, September 19, 2012) [33], *Levofloxacin Hydrochloride* (in the 56th ADRIB, August 2, 2013) [34] and *Cefazolin* (in the 59th ADRIB, January 26, 2014) [35] were detected with IC_025_ by stratification method. It could be noted that these DRUGs were reported in a large order of magnitude, but the frequencies of the DECs corresponding to them were very small. Therefore, when OE ratios were calculated based on disproportionality method, the expected value increased while the observed value decreased, which resulted in the decreased OE ratios and masked signals. Data masking effect was particularly evident to *Levofloxacin Hydrochloride* in Table 4. By stratification, the reports of the drug were scatter into many clusters, in which the frequency declined significantly. As a result, 61 DECs related to it were newly unmasked. Similarly, 5 DECs related to *Potassium Magnesium Aspartate* and 26 DECs related to *Cefazolin* were unmasked by stratification.

**Table 4:** Signal detection results related to *Potassium Magnesium Aspartate*, *Levofloxacin Hydrochloride* and *Cefazolin*

Drug (frequency)	ADR	DECs’ frequency	IC_025_ of non-stratification	IC_025_ of stratification
*Potassium Magnesium Aspartate* (2043)	*pruritus*	65	−2.42	1
	*anaphylactoid reaction*	63	−0.67	0.94
	*headache*	54	−1.17	0.64
	*dyspnoea*	40	−0.25	0.48
	*cyanosis*	11	−0.59	0.06
*Levofloxacin Hydrochloride* (111,335)	*larynx oedema*	69	−0.32	4.04
	*Infusion reaction*	66	−0.93	3.76
	*skin discolouration*	41	−3.46	3.7
	*vesicular rash*	54	−1.46	3.02
	*tachycardia*	66	−2.37	2.53
	*agitation*	75	−0.52	2.37
	*asthma*	29	−2.2	2.3
	*application site pain*	114	−0.39	2.04
	*arrhythmia*	26	−3.04	1.61
	*ecphysesis*	121	−1.18	1.38
*Cefazolin* (15,105)	*injection site rash*	67	−0.12	2.27
	*larynx oedema*	18	−0.21	1.47
	*ecphysesis*	44	−0.17	1.41
	*injection site pruritus*	30	−1.95	1.39
	*haematuria*	22	−0.99	1.07
	*oedema periorbital*	31	−0.61	1.06
	*anaesthesia local*	57	−1.21	1.05
	*cutireaction*	43	−1.18	1.04
	*diarrhoea*	72	−3.34	0.9
	*sweating increased*	79	−0.48	0.9

## Discussion

Data masking or competition bias is an inborn defect in disproportionality analysis which depends on OE ratio to highlight DECs. Some measures can be adjusted to minimize any undue influence on the ADR reporting rate of covariates by performing stratification according to a set of common potential confounders [28]. However, these adjusted methods still result in data masking as ignoring frequency differences between ADEs. To reduce the impact on OE ratios, this pilot study mainly focuses on minimizing the data masking effect in signal detection by stratification based on clustering. The study dataset is stratified into some clusters according to the sequence of “DRUGs-ADRs-DECs” and signal detection is conducted by the IC method of disproportionality for each cluster respectively. All highlighted DECs are collected to evaluate unmasking performance of stratification based on the reference database and ADRIB. The specific number of clusters is determined by data distribution characteristics, and stratification is performed by *K-means* clustering algorithm step by step. Such processes can avoid the subjective decision existing in other stratification methods.

In our study dataset, there are more than one million reports where the frequencies of DECs vary greatly for various reasons, such as the frequent uses of drugs, the differences of drug side effects or even the individual selective reporting. The over-reported DRUGs, ADRs and DECs are more likely to mask some specific DECs which are less reported but actually true signals. The frequency distribution of reports in each cluster is smoothed by stratification, which is different from other stratification methods where the whole dataset is stratified into several strata according to confounding factors such as gender, age, region, etc.

TP signals increase from 6853 to 10,336 with stratification, which means a significant increase in the number of positive signals. These signals include 5046 TP signals and 5290 FP signals. The increase in the signals identified by our method is due to the fact that all high frequency drugs or ADEs are divided into different clusters, which reduces the possibility of the related low frequency DECs being masked. Among 5046 TP signals, 1656 signals (32.82%) are not detected by non-stratification, which fully proves our method can better minimize data masking. While FP signals increase from 3114 to 5290, which means more workload is need in signal evaluation for pharmacovigilance.

There are some limitations in this study. First, 2 years of spontaneous reporting data may not fully represent total data in CSRD. Then, as the gold standard for performance evaluation of signal detection, the reference database is extracted from drug product labeling manually, and the omissions or errors are unavoidable. Meanwhile, only the IC method is adopted for signal detection. The other methods of disproportionality analysis, such as PRR and MHRA, are not tried to verify the proposed method. These limitations above may lead to uncertain impact on the experimental results.

## Conclusion

This paper proposes a stratification method based on clustering for the minimization of masking in signal detection. All reports of 2 years in the CSRD are stratified into some clusters, among which DRUGs, ADRs or DECs are of the similar order of magnitude in frequency. Experimental results show that better performance for unmasking signals is obtained with stratification. Owing to the decline of *Precision*, we suggest that this method can be used in parallel to non-stratification method rather than replacing it.

## Data Availability

This research comes from a project which the CFDA commissioned me and other authors to undertake. All ADR spontaneous reporting data in this study is licensed by the CFDA. The data is not publicly available due to the policy of confidentiality of the CFDA but are available from the corresponding author on reasonable request and with permission of the CFDA.

## Abbreviations

ADEs: Adverse drug events
ADRIB: Adverse drug reaction information bulletin
ADRs: Adverse drug reactions
CFDA: China food and drug administration
CSRD: Chinese spontaneous reporting database
DECs: Drug-event combinations
FN: False negatives
FP: False positives
IC: Information component
MHRA: Medicines and Healthcare Products Regulatory Agency
OE: Observed-to-expected
PRR: Proportional reporting ratio
ROR: Reporting odds ratio
SRS: Spontaneous reporting system
TN: True negatives
TP: True positives
